# Exploring the Influence of Demographic Factors and Flourishing on Workplace Distractions: A Cross-Country Analysis

**DOI:** 10.1155/2024/2431300

**Published:** 2024-09-26

**Authors:** Emad Shdaifat, Tamadur Shudayfat, Noha Al-Shdayfat, Nora Alotaibi, Mona Alduhaileb

**Affiliations:** ^1^ Community Nursing Department College of Nursing Imam Abdulrahman Bin Faisal University P.O. Box 1982, Dammam, Saudi Arabia; ^2^ Community and Mental Health Nursing Department Faculty of Nursing Al al-Bayt University P.O. Box 130040, Mafraq, Jordan; ^3^ Community and Mental Health Nursing Department Zarqa University, Zarqa, Jordan; ^4^ Nursing Professional Development Department Dammam Medical Complex, Dammam, Saudi Arabia

**Keywords:** cross-country analysis, flourishing, Jordan, nurses, Saudi Arabia, workplace distractions

## Abstract

**Objective:** To explore the influence of demographic factors and flourishing on workplace distractions in a cross-country analysis of Saudi Arabia and Jordan.

**Methods:** This cross-sectional comparative study was conducted in two government hospitals: one in eastern Saudi Arabia and the other in northern Jordan. Data were collected from the nurses using convenience sampling. The required sample size was determined using the G∗Power software, with a target of 242 nurses per country, resulting in 484 participants. Ultimately, the final sample consisted of 437 nurses: 222 from Saudi Arabia and 215 from Jordan. Two online instruments were used to collect data on the distractions and flourishing.

**Results:** The study found that “using the Internet” and “using the phone” were the most time-consuming distractions among nurses, while “watching TV” was the least time-consuming. There were significant associations between demographics and distractions, with participants from Saudi Arabia having lower distraction scores than participants from Jordan. Younger participants were more distracted than older participants, whereas male participants were more distracted than female participants were. Smokers and individuals with less expertise exhibit higher levels of distraction. Furthermore, degree of education was associated with higher levels of distraction. Nurses in Saudi Arabia experience a much greater loss of productivity due to distractions than their Jordanian counterparts. Regression analysis revealed that experience, “Mental and Physical Health” domain scores, smoking status, and educational level all highly predicted distraction levels among Saudi Arabian and Jordanian nurses, accounting for approximately 9.6% of the differences in distraction.

**Conclusion:** In this cross-country study on workplace distractions among Saudi and Jordanian nurses, “using the Internet” and “using the phone” emerged as the most time-consuming distractions. Younger age, male sex, smoking, and less experience are associated with higher distraction levels. Additionally, higher education levels were linked to increased distraction. Implementing employee flourishing activities can help reduce distractions and enhance productivity. This study offers valuable insights into improving nurses' performance and well-being.

## 1. Introduction

Distraction is a constant challenge in fast-paced modern work environments that interferes with activities and leads to consequences, such as medical errors, conflicts, delays in treatment, and reduced healthcare productivity [1–4]. Distractions take someone's focus away from their current task [5]. Nurses must pay attention while managing a variety of tasks in a fast-paced atmosphere in order to provide high-quality care. Every action can have a different result and disastrous effects. Therefore, distractions have been extensively researched in the healthcare field [6]. Complex skills are needed to handle these disruptions because distractions can lead to nurses losing focus, which raises the possibility of patient safety problems [7, 8]. According to Sørensen and Brahe [9], nurses are frequently questioned about their work and are asked for patient information. Other healthcare professionals, nursing staff, phones, patients, family members, visitors, and self-interruptions are primary causes of distraction [9, 10]. Westbrook et al. observed 98 nurses who prepared and administered 4,271 drugs to 720 patients in an Australian hospital. According to study findings, a nurse's probability of making a medication error increased by 12.7% for every interruption, and this risk tripled when a nurse was interrupted six times [4]. According to a study by Hall et al. [11], which examined 13,025 distractions encountered by medical and surgical nurses in 36 units from nine hospitals, 90% of distraction-related errors led to treatment delays or a loss of attention or concentration. These mistakes occur frequently during paperwork, medical administration, procedures, or assessments related to patient care.

Flourishing, encompassing well-being and efficient work, plays a crucial role in employee satisfaction. It represents a state of positive health, characterized by high levels of emotional, psychological, and social well-being [12]. It encompasses happiness, resilience, commitment, and determination and contributes to overall well-being. Positive health behaviors and outcomes [13], job satisfaction [14], and optimal biomarkers of inflammation [15] have all been linked to flourishing in previous research. A recent study involving resident physicians found that flourishing was positively correlated with resilience and coping and negatively correlated with emotional exhaustion and negative affect [16]. Flourishing can help nurses overcome adversity, such as hospitalized patients who are incurable, by giving them confidence to act quickly to preserve patients' lives, hope that every patient will recover, and great optimism for patient healing [17].

Successful employees experience fewer difficulties at work, enjoy autonomy, receive better rewards, and receive more support than their less successful colleagues [18]. The work environment also influences well-being, with stimulating work promoting well-being and uninteresting environments having a negative impact on overall flourishing [19].

Interruptions that require attention, such as phone calls, differ from distractions and irrelevant inputs, which must be ignored [20–22]. An average of 86 interruptions per day affect concentration and productivity [23]. Despite the importance of well-being, few studies have examined its relationship with distraction [24]. Internal (malaise and fatigue) and external (personal contact, emails, and calls) factors contribute to distractions and cause errors, accidents, poor work quality, stress, anger, emotional fatigue, and reduced job satisfaction and engagement [25].

Healthcare facilities, which are particularly prone to distractions, have recently received attention due to potential errors and lack of focus on important tasks [7, 8, 20]. Although previous research has examined the types of distractions, few studies have focused on nursing staff distraction costs and related variables. This study fills this gap by comparing distraction costs among Saudi Arabian and Jordanian nurses, identifying the causes, examining work-related factors, and examining the role of flourishing in reducing productivity. Demographic impacts were also examined to provide cross-national insights into improving employee well-being and healthcare efficiency. In this study, we aimed to expand the existing knowledge base in this field by examining the causes, effects, and potential mitigating variables of nursing distractions. It seeks to provide useful information that can direct practice and policy, ultimately enhancing staff well-being and patient care.

## 2. Methods

### 2.1. Site, Setting, and Design

This comparative cross-sectional study was conducted in the eastern region of Saudi Arabia and northern Jordan, specifically focusing on government hospitals that fall under the Ministry of Health (MOH). The data for this study were gathered from nurses working in various units in these hospitals. The study was conducted in a government hospital located in the Eastern Province of Saudi Arabia. This region is renowned for its diverse population and rapid urbanization, resulting in a mix of urban and rural healthcare settings. In Jordan, the data were collected from a government hospital in the northern region, which predominantly serves rural and semiurban populations. This provides valuable insights into healthcare delivery in less urbanized settings. These locations were chosen to encompass a wide range of sociodemographic characteristics and healthcare environments, thereby enhancing the generalizability of the study's findings. An age dichotomy of 40 years was chosen based on the available literature, which indicates that age 40 is a significant milestone in the careers and lifestyles of nurses. This cutoff aids in distinguishing between younger and older participants in terms of their workplace experiences and vulnerability to distractions. Experience in this study pertains to the duration that nurses have devoted to their profession, which is being considered to assess its influence on workplace distractions and productivity. BMI data were collected to explore their correlation with workplace distractions, as prior research has revealed that physical health can affect cognitive function and productivity, thus establishing BMI as a pertinent variable.

### 2.2. Sampling and Sample Size and Calculation

The population sample consisted of nurses, and a convenience sample was used. Nurses working in government hospitals in both countries were invited to participate in the study. This study primarily focused on governmental hospitals that fall under the purview of the MOH as they exemplify the wider healthcare system and abide by regulatory standards imposed by the MOH. The required sample size was calculated using the G∗Power software, resulting in 242 nurses estimated for each country, assuming an effect size of 0.3 and alpha equal to 0.05. An additional 20% was added to account for potential nonresponse. In total, 484 nurses were recruited to ensure adequate response rates.

### 2.3. Instruments

Two instruments were used in the study. The first instrument focuses on distractions, gathering data on the frequency and duration of various types of distractions to discern patterns [26]. Participants reported how often they had engaged in each type of distraction in the previous week and how much time it took them to complete each distraction. The total duration for each participant was calculated by multiplying the distraction time with the frequency of each week. The participants were surveyed regarding the distractions they encountered in their workplace using a comprehensive questionnaire. For instance, one of the questions inquired, “How frequently did you experience distractions during the working day, and for how long did these distractions persist?” Participants provided specific information pertaining to the frequency and duration of distractions across a range of activities such as conversing with colleagues, utilizing the phone and Internet, scheduling personal appointments, engaging in online shopping, making bill payments, receiving visits from family or friends, taking breaks for snacks or smoking, organizing clutter, participating in gossip, extending lunch breaks, and attending meetings.

The second instrument is the Flourishing Index. There are 12 elements in each of the six domains: Happiness and Life Satisfaction, Mental and Physical Health, Meaning and Purpose, Character and Virtue, Close Social Relationships, and Financial and Material Stability. Each point was scored on an 11-point scale ranging from 0.0 to 10.0. The scores for each of the first six domains were added, with the extreme categories flagged and aligned to higher scores indicating more positive responses. High scores indicated that people had strong opinions about themselves when it came to human flourishing. The questionnaire covered various domains of well-being, with the participants rating their experiences on an 11-point scale. For instance, participants were instructed to assess their overall life satisfaction by envisioning a ladder with steps ranging from 0 at the bottom to 10 at the top, representing the best and worst possible life scenarios, respectively. The participants were also asked to evaluate their general level of happiness using the same scale. In addition, participants were asked about their perceptions of their physical health, with responses ranging from 0 to 10 [27, 28]. The survey was conducted using the online survey platform, QuestionPro (https://www.questionpro.com). QuestionPro was chosen because of its user-friendly interface, secure data-handling capabilities, and proficiency in managing large-scale surveys across various geographical regions. This platform enabled us to effectively engage a broad range of nurses in Saudi Arabia and Jordan, thereby ensuring a rigorous and uniform data collection process. Furthermore, QuestionPro's functionalities, including real-time data analytics and customizable survey templates, have facilitated extensive data management and analysis. The survey was conducted in Arabic.

### 2.4. Pilot Study

Two key pilot studies were conducted to assess the questionnaire's construct validity in different settings. In a Jordan pilot study involving 40 nurses, Pearson's correlation analysis identified questions as valid if their *R* value exceeded 0.3120, as determined by the critical score table. Improvements in the participants' clarity were made through careful adjustments to two negligible points. The internal consistency assessment conducted using Cronbach's alpha analysis highlighted robust coherence, with an *r* value between 0.411 and 0.785 and a commendable Cronbach's alpha coefficient of 0.825. Likewise, the Saudi pilot study involving 27 nurses relied on Pearson correlation analysis for item construct validity. Questions were considered valid if their *R* value exceeded 0.381, based on the cutoff value of the correlation analysis table. The results were both meaningful and valid and were confirmed by reliable Cronbach's alpha analysis, with *r* values ranging from 0.524 to 0.866, yielding a remarkable Cronbach's alpha coefficient of 0.9224 [29]. Both pilot studies confirmed the questionnaire's construct validity and provided valuable insights into its clarity and cultural appropriateness. Minor adjustments were made based on participant feedback and quantitative analysis to improve clarity and relevance. The measures of distraction used in this study were specifically developed to address the gaps identified in the literature, and the detailed rationale for their development and psychometric testing procedures is included in the revised manuscript. These refinements ensured that the questionnaire is robust and culturally relevant to the main study.

### 2.5. Ethical Consideration

The study protocol was approved by the Institutional Review Board of Imam Abdulrahman Bin Faisal University in Saudi Arabia (IRB-2022-04-275) and Al al-Bayt University in Jordan (Protocol no. 10/2021/2022). The participants were informed about the purpose, significance, and benefits of the study and provided with an information sheet. The participants emphasized that their involvement was voluntary and that there would be no physical or mental harm incurred from the study. Informed consent was obtained from all the participants. The collected data were treated as confidential, and the research team was solely responsible for its management.

### 2.6. Cost Analysis

The cost analysis conducted in this study aimed to examine lost productivity, which was measured by calculating the average minutes lost per week per nurse for each distractor. Two measures, the mean and interquartile range (IQR), were used to represent the distribution of lost minutes. The mean provided an average estimate of the time lost, while the IQR captured the central spread of the data, offering a robust measure of the central tendency that was less affected by outliers. The lost minutes per nurse were then multiplied by the pay per minute for each nurse in both countries, resulting in monetary loss. By quantifying the time spent on distractors, the calculation of productivity loss allows for the direct conversion of time loss into monetary amounts. This approach provides a practical perspective on the economic impacts of distractors. To evaluate the cost implications of workplace distractions, economic calculations incorporated the reported time allocated to distractions and the average wage rates for nurses in each country. The economic impact was determined by estimating the monetary value of productivity loss.

### 2.7. Data Analysis

Data were stored and analyzed using SPSS version 22. Microsoft Excel was used to calculate the cost of the lost productivity. Continuous data are presented as means and standard deviations, while categorical variables are presented as frequencies and percentages. The internal consistency of the scales used in this study was tested using reliability analysis, and Cronbach's alpha coefficients were calculated. Independent *t*-tests and ANOVA were used to determine differences between groups. Pearson's correlation coefficients were used to assess the relationships between variables. Multiple linear regression was used to predict the cost of the distractions. Statistical significance was set at *p* of less than 0.05.

### 2.8. Validity and Reliability

The study had a total response rate of 53.1% in both countries. Of the initial sample of 484 invited participants (242 from each country), 437 nurses completed the surveys. Specifically, 222 nurses from Saudi Arabia and 215 from Jordan participated in this study. Nineteen participants were excluded from the analysis due to missing data, with one participant from Saudi Arabia and 18 from Jordan being affected. Additionally, 18 participants were excluded owing to a lack of variance, and eight participants with more than 20% missing data were removed to prevent bias. Imputation filled the gaps for variables with values less than 20%. The alpha value (0.678–0.918) confirmed the internal consistency of the scale. Three outliers were identified using Mahalanobis distances, eliminated at the 5% significance level, and influence integrity was preserved. After these steps, the final sample size for the analysis was 437 participants to increase the reliability. Transformations ensured the normality of the dependent variables. Validation of the distraction and flourishing scales using Pearson's correlation showed significant relationships (*p* < 0.001), confirming the robustness and reliability of the study in subsequent statistical analyses.

## 3. Results

The study had an overall response rate of 53.1%, with 437 participants completing the survey. Of these, 222 were from Saudi Arabia and 215 were from Jordan. The majority of the participants were 40 years old (80.8%), female (79.2%), and nonintensive care professionals (73.2%) and had 20 years of experience (87.4%). The proportion of nonsmokers was 79.4%, 70.3% were married, 43.7% had a diploma, and 47.1% had a BSc degree. The BMI distribution was 2.1% underweight, 36.6% normal weight, 38.7% overweight, and 22.7% obese. These demographic data provided valuable insights into the composition of the participants and improved our understanding of the study results (Table 1).

### 3.1. Cronbach's Alpha

Cronbach's alpha coefficient of the flourishing scale was 0.896, indicating a high level of internal consistency. A total of 437 participants were included in this study, with 50.8% from Saudi Arabia and 49.2% from Jordan.

Table 2 shows the data on various distractions and weekly time lost by nurses. The highest average minutes lost occurred when using the Internet (mean = 62.9), followed by telephone (mean = 50.6), and the least when watching television (mean = 9.5). The total distraction category showed that nurses lost an average of 378.9 min to distractions, with a median of 194.0 min.

Table 3 highlights the notable differences in distraction levels between participants from Saudi Arabia and Jordan in the different categories. Participants from Saudi Arabia reported lower total distractions (mean = 308.6) than those from Jordan (mean = 455.2), indicating a significant disparity in the total distracted experiences (*p*=0.014). In particular, there was a significant difference in the occurrence of smoking/snacking breaks between the two groups. Participants from Saudi Arabia (mean = 14.1) reported significantly fewer distractions in this category than did participants from Jordan (mean = 33.9, *p*=0.001). These results suggest that people from Saudi Arabia generally experience fewer distractions and take fewer smoke/snack breaks than their Jordanian counterparts.

The lost productivity of all nurses in Saudi Arabia due to all types of distractions was estimated at 308.6 min per week. This equates to a monetary loss of 84.22947 USD per week, considering a payment rate of 0.2223 USD per minute. Likewise, the lost productivity for all nurses in Jordan due to all types of distractions was calculated at 455.2 min per week. This equates to a monetary loss of 40.33072 USD per week with a payment rate of 0.0886 USD per minute.

Table 4 shows the demographic influences of the distractions. Participants from Saudi Arabia had a lower average distraction score (308.6) than their Jordanian counterparts (455.2) (*p*=0.014). Age under 40 years correlated with a higher mean distraction score (444.6) than age over 40 years (113.8) (*p*=0.001). Men had a higher average distraction score (523.3) than women (343.4) (*p*=0.015). Experience under 20 years was associated with a higher mean distraction score (426.9) than experience over 20 years (62.1) (*p*=0.001). Smoking status influenced distractions: Nonsmokers scored 342.4, while smokers had a higher average score of 529.0 (*p*=0.012). Differences in educational levels were evident; those with a diploma scored 277.9 points, BSc holders scored 436.6, and MSN or PhD holders scored the highest at 586.9. There was a significant difference in the distraction scores between graduates and college graduates (*p*=0.004).

The analysis found that age was a significant factor affecting flourishing scores, with those aged 40 years and younger having lower average scores in the areas of Happiness and Life Satisfaction, Mental and Physical Health, and Meaning and Purpose (*p*=0.023, *p*=0.024, and *p*=0.022). Conversely, those over 40 years of age tended to have a higher level of prosperity. Gender-specific scores in the Mental and Physical Health domains (*p*=0.012) indicated sex differences in flourishing. Experience also played a role; participants over 20 years of age showed higher scores in all areas (*p*=0.002, *p*=0.018, *p*=0.028, and *p*=0.001), indicating increased flourishing with greater experience. However, education level, smoking habits, marital status, type of specialty, and body mass index did not show any significant influence on the level of success. No significant differences were observed between graduate and college graduates, smokers and nonsmokers, marital status, discipline, or body mass index categories, implying limited or negligible effects on overall flourishing behavior (Table 5).

Table 6 shows the correlations between the amount of distraction in minutes per week and different domains of flourishing. These correlations provide insight into the strength and direction of the relationships between these variables. These results suggest a moderately negative association between distraction and several flourishing domains. In particular, higher levels of distraction correlated significantly with lower levels of flourishing in the Happiness and Life Satisfaction domains (*r* = −0.172, *p*=0.001), Mental and Physical Health domains (*r* = −0.202, *p*=0.001), and Close Social Relationships domains (*r* = −0.142, *p*=0.003). However, the correlations between distraction and the Meaning and Purpose (*p*=0.112), Character and Virtue (*p*=−0.456), and the Financial and Material Stability (*p*=−0.313) domains were not statistically significant.

The model summary showed that approximately 9.6% of the distraction variation was explained by predictors, accounting for approximately 10.4% of the differences. The Durbin–Watson statistic (1.858) suggests no systematic errors in the predictions. The ANOVA table shows the statistical significance of the model (*F* = 12.537, *p* < 0.001), indicating that the predictors collectively influenced distraction. Stepwise regression identified significant predictors including experience (negative association, *p*=0.001), mental and physical health (inverse association, *p*=0.001), smoking (positive association, *p*=0.020), and educational level (higher levels associated with distraction, *p*=0.025). In summary, experience, mental/physical health, smoking, and education predicted distraction, with more experienced, healthier, and better-educated participants as well as smokers exhibiting varying levels of distraction (Table 7).

## 4. Discussion

This study aimed to explore the impact of demographic factors and flourishing on workplace distraction among nurses in Saudi Arabia and Jordan. The results demonstrated that “using the Internet” and “using the phone” were the most time-consuming distractions, consistent with previous research that has identified these as common sources of interruption in healthcare settings [7, 30]. The extensive utilization of technology in contemporary healthcare, although crucial for communication and information management, also poses significant challenges. These distractions have the potential to disrupt the workflow, diminish efficiency, and compromise patient safety by diverting attention away from critical tasks.

The demographic analysis revealed that younger, male, and less experienced nurses, as well as those who smoked, reported higher levels of distraction. These findings are consistent with previous studies that indicate that younger and less experienced nurses may be more susceptible to distractions due to a lack of developed coping strategies and greater reliance on technology for both work-related and personal tasks [7]. The increased levels of distraction among male nurses may be attributed to varying stress responses or social factors that influence their interactions with technology. Smoking, often used as a coping mechanism for stress, appears to be associated with higher levels of distraction, suggesting that nurses may be more inclined to take breaks or divert their attention during work hours.

An association between educational level and distraction was observed, with nurses with higher education levels reporting a greater incidence of distraction. This correlation can be attributed to the heightened cognitive demands placed on nurses with advanced education, who frequently undertake additional responsibilities necessitating multitasking, thereby augmenting their vulnerability to potential distractions. These findings highlight the intricate nature of the nursing work milieu, wherein personal attributes and professional obligations interact to shape the degree of distraction experienced.

The cross-country comparison conducted in this study revealed that despite reporting fewer distractions overall, Saudi nurses encountered a more significant decrease in productivity than their Jordanian counterparts. This finding, which seems contradictory, implies that, although Saudi nurses may be more skilled in limiting the number of distractions, the distractions they encounter may have a more profound influence on their workflow. This outcome can potentially be ascribed to cultural or systemic factors within the Saudi healthcare system that exacerbate the effects of distractions such as increased workloads or more stringent performance expectations. Conversely, Jordanian nurses may employ more effective strategies to manage distractions, resulting in lower perceived impact on productivity.

The finding regarding the substantial influence of flourishing, specifically on both mental and physical well-being, in predicting distraction levels is essential. Nurses who exhibit better health reports experience fewer distractions, emphasizing the crucial role of well-being in sustaining concentration and productivity. This observation is in line with previous research which indicates that fostering mental and physical health within the workplace can heighten employee performance and decrease the occurrence of errors [31, 32]. Consequently, initiatives designed to promote flourishing, such as stress management programs, mindfulness training, and activities promoting physical wellness, hold significant potential for mitigating the detrimental effects of distractions and ultimately enhancing overall nurse performance.

These findings have important implications for patient safety. It is well established that distractions in healthcare settings increase the risk of errors, which can ultimately lead to grave consequences for patient outcomes [4]. Through the identification of pivotal predictors of distraction, this study offers valuable insights into the development of targeted interventions with the objective of minimizing distractions. For instance, training programs that bolster coping skills among younger and less experienced nurses, as well as initiatives that foster healthy lifestyles and stress management, hold promise in this regard.

The cultural disparities observed between Saudi and Jordanian nurses underscore the need to employ context-specific methodologies to effectively address workplace distractions. Interventions should be customized to account for the distinctive cultural and systemic elements that shape nursing practices in each country. For instance, strategies that demonstrate efficacy in Jordan may require adaptation to align with the distinct work environments prevalent in Saudi Arabia.

In conclusion, this study contributes to academic knowledge on the correlation between demographic factors, flourishing, and workplace distractions in the nursing work environment. The findings indicate that mitigating distractions has the potential not only to enhance the well-being and performance of nurses but also to promote patient safety. Further research should focus on studying the long-term effects of these distractions and evaluating the efficacy of different intervention strategies across various healthcare settings. By addressing these challenges, healthcare systems can provide better support to nursing staff, ultimately resulting in improved quality of care and patient outcomes.

This study had several limitations. The study's use of a specific hospital sample in Saudi Arabia and Jordan restricts the generalizability of the findings to other regions or healthcare settings. Convenience sampling may introduce selection bias, as participants may not adequately represent the broader nursing population. The reliance on self-reported data introduces a response bias, which may result in inaccuracies due to social desirability or recall issues. Additionally, the cross-sectional design does not allow for causal inferences, capturing only a single point in time and limiting the establishment of temporal relationships. By focusing solely on nurses, the applicability of the results to other healthcare professionals or industries is limited.

The assumptions made in an economic analysis of productivity loss may oversimplify the complex factors that influence workplace efficiency, potentially distorting impact estimates. Furthermore, the exclusion criteria and removal of outliers, although necessary, may have introduced bias by excluding data that may have influenced the results. Finally, the instruments used, while validated, may not fully capture the cultural nuances or specific workplace challenges faced by nurses in different countries. Despite these limitations, this study offers valuable insights into workplace distractions among nurses, thus informing future research.

## 5. Nursing Implications

Understanding the factors that hinder the influence of demographic factors and flourishing on workplace distractions among nurses in Jordan and Saudi Arabia will assist policymakers in both countries, governments, NGOs, and other stakeholders in creating targeted policies and programs. These initiatives will prepare an appropriate work environment and help reduce sources of workplace distractions. Healthcare organizations should manage their cell phone usage to minimize distractions by establishing effective and practical usage policies. Additionally, we recommend orientation programs for novice nurses that tailor behaviors to reduce workplace distractions, including smoking and frequent cell phone use. In addition, we recommend the implementation of policies that restrict cell phone use in clinical settings.

## 6. Conclusions

This study sheds light on the critical impact of nurse distractions on well-being, including happiness, mental and physical health, and social relationships. Factors such as age, sex, experience, smoking, and education have a significant influence on these distractions. Our findings underscore the economic ramifications of nurse distraction in Saudi Arabia and Jordan. Regression analysis identified experience, health status, smoking, and education as the key predictors of distraction levels. Despite limitations, such as convenience sampling and potential self-report bias, our results provide valuable insights into the adverse effects of nurse distractions and their economic implications. This study emphasizes the necessity for targeted interventions and policies to mitigate distractions and enhance nurses' well-being. Future research should investigate the longitudinal effects of these distractions and assess the effectiveness of specific interventions in diverse healthcare settings.

## Figures and Tables

**Table 1:** Demographic characteristics of participants.

**Variable**	**Category**	**Frequency**	**Percent**
Residency	Saudi	222	50.8
	Jordan	215	49.2

Age	≤ 40	353	80.8
	> 40	84	19.2

Gender	Male	91	20.8
	Female	346	79.2

Specialty	Critical	117	26.8
	Noncritical	320	73.2

Experience	≤ 20	382	87.4
	> 20	55	12.6

Smoking	No	347	79.4
	Yes	90	20.6

Marital status	Single	104	23.8
	Married	307	70.3
	Divorce or widowed	26	5.9

Educational level	Diploma	191	43.7
	BSc	206	47.1
	MSN or PhD	40	9.2

BMI	Underweight	9	2.1
	Normal	160	36.6
	Overweight	169	38.7
	Obesity	99	22.7

**Table 2:** Type of distraction and minutes loss per week among nurses.

**Type of distraction**	**Mean (SD)**	**Median (IQR)**	**Minimum**	**Maximum**
Use the Internet	62.9 (155.7)	15.0 (44.5)	0	961
Use the phone	50.6 (131.3)	12.0 (32.0)	0	961
Discussions with coworkers, patients, or work acquaintances	31.4 (84.7)	12.0 (26.0)	0	961
Meeting	25.7 (82.6)	4.0 (21.0)	0	961
Clutter	25.0 (89.5)	2.0 (15.0)	0	961
Smoke and snack breaks	23.8 (61.1)	6.0 (24.5)	0	961
Gossip	22.5 (100.7)	1.0 (11.0)	0	961
Organizing and planning for personal time	20.6 (37.2)	9.0 (21.0)	0	341
Daydream	19.6 (79.7)	1.0 (9.5)	0	930
Taking too long of a lunch with colleagues	18.9 (57.6)	2.0 (21.0)	0	961
Pay personal bills	17.8 (79.7)	2.0 (8.0)	0	961
Shop from work by catalog, phone, or Internet	16.3 (55.0)	1.0 (11.0)	0	961
Play computer games	12.9 (74.1)	1.0 (3.0)	0	961
Leisure reading (e.g., books and magazines)	10.8 (50.0)	1.0 (5.0)	0	961
Visits at work from family, friends, or others	10.4 (24.2)	1.0 (7.5)	0	248
Watch TV	9.5 (25.8)	1.0 (4.0)	0	248
Total distractions	378.9 (638.0)	194.0 (324.5)	0	6315

**Table 3:** The difference in distraction between Saudi and Jordan.

**Distraction/country**	**Saudi**	**Jordan**	**95% CI**	**Sig**
	**Mean (SD)**	**Mean (SD)**		
Discussions with coworkers, patients, or work acquaintances	37.9 (115.2)	24.6 (29.1)	(−2.4, 29.0)	0.096
Use the phone	49.9 (140.1)	51.3 (122.0)	(−26.1, 23.4)	0.916
Use the Internet	74.8 (191.8)	50.6 (105.5)	(−4.8, 53.2)	0.102
Organizing and planning for personal time	20.1 (40.7)	21.0 (33.4)	(−7.9, 6.1)	0.803
Shop from work by catalog, phone, or Internet	18.0 (68.9)	14.5 (35.4)	(−6.8, 13.9)	0.504
Pay personal bills	19.9 (73.2)	15.8 (86.1)	(−11.0, 19.1)	0.601
Visits at work from family, friends, or others	8.2 (17.5)	12.6 (29.5)	(−8.9, 0.2)	0.061
Daydream	20.4 (95.4)	18.7 (59.5)	(−13.3, 16.7)	0.828
Leisure reading (e.g., books and magazines)	13.6 (67.9)	7.8 (18.3)	(−3.6, 15.0)	0.226
Play computer games	15.5 (91.7)	10.4 (50.0)	(−8.9, 19.0)	0.478
Watch TV	9.9 (25.0)	9.1 (26.8)	(−4.1, 5.7)	0.746
Smoke & snack breaks	14.1 (24.7)	33.9 (82.4)	(−31.4, −8.3)	0.001
Clutter	25.3 (99.5)	24.7 (78.1)	(−16.2, 17.5)	0.938
Gossip	16.1 (91.9)	29.3 (108.9)	(−32.1, 5.8)	0.173
Taking too long of a lunch with colleagues	15.3 (67.1)	22.7 (45.9)	(−18.2, 3.5)	0.183
Meeting	24.0 (76.6)	27.4 (88.5)	(−19.0, 12.1)	0.667
Total distractions	308.6 (648.7)	455.2 (593.8)	(−263.7, −29.4)	0.014

**Table 4:** Demographic characteristics difference related to distraction.

**Variable**	**Category**	**Mean (SD)**	**95% CI**	**Sig**
Residency	Saudi	308.6 (648.7)	(−263.7, −29.4)	0.014
	Jordan	455.2 (593.8)		

Age	≤ 40	444.6 (594.1)	(184.6, 477.1)	0.001
	> 40	113.8 (686.1)		

Gender	Male	523.3 (575.8)	(35.8, 324.1)	0.015
	Female	343.4 (633.8)		

Specialty	Critical	467.7 (645.2)	(−14.8, 251.2)	0.081
	Noncritical	349.5 (616.7)		

Experience	≤ 20	426.9 (609.8)	(190.6, 539.1)	0.001
	> 20	62.1 (647.9)		

Smoking	No	342.4 (634.4)	(−331.3, −42.0)	0.012
	Yes	529.0 (571.4)		

Marital status	Single	508.7 (578.9)	(395.6, 621.9)	0.056
	Married	344.8 (633.3)	(273.7, 415.9)	
	Divorce or widowed	301.1 (669.9)	(30.5, 571.7)	

Educational level	Diploma	277.9 (639.3)	(186.7, 369.2)	0.004^a^
	BSc	436.6 (610.5)	(352.6, 520.7)	
	MSN or PhD	586.9 (562.0)	(407.2, 766.7)	

BMI	Underweight	289.8 (209.1)	(129.1, 450.6)	0.542
	Normal	423.1 (614.9)	(326.8, 519.4)	
	Overweight	386.9 (599.8)	(295.8, 478.0)	
	Obesity	311.2 (706.9)	(170.2, 452.2)	

^a^Tukey HSD: diploma versus BSc (*p*=0.030); diploma versus MSN or PhD (*p*=0.012).

**Table 5:** Demographic characteristics difference related to flourishing.

**Variables/domains**		**Happiness and Life Satisfaction**		**Mental and Physical Health**		**Meaning and Purpose**		**Character and Virtue**		**Close Social Relationship**		**Financial and Material Stability**	
**Variable**	**Category**	**Mean (SD)**	**Sig**	**Mean (SD)**	**Sig**	**Mean (SD)**	**Sig**	**Mean (SD)**	**Sig**	**Mean (SD)**	**Sig**	**Mean (SD)**	**Sig**
Residency	Saudi	12.9 (4.6)	0.121	13.9 (4.4)	0.370	15.5 (3.9)	0.657	15.7 (3.5)	0.772	15.0 (4.3)	0.051	11.9 (5.3)	0.520
	Jordan	12.3 (4.2)		14.3 (3.6)		15.4 (3.3)		15.8 (3.2)		14.2 (4.0)		11.6 (4.7)	

Age	≤ 40	12.4 (4.3)	0.023	13.9 (4.1)	0.024	15.3 (3.7)	0.022	15.7 (3.3)	0.361	14.2 (4.2)	0.001	11.7 (4.9)	0.290
	> 40	13.6 (4.7)		15.0 (3.7)		16.3 (3.3)		16.1 (3.6)		16.1 (3.8)		12.5 (5.5)	

Gender	Male	12.8 (4.5)	0.630	14.9 (3.3)	0.012	16.0 (2.9)	0.077	16.3 (2.8)	0.051	14.8 (3.7)	0.601	11.9 (5.0)	0.736
	Female	12.6 (4.4)		13.9 (4.2)		15.3 (3.8)		15.6 (3.5)		14.5 (4.3)		11.7 (5.0)	

Specialty	Critical	12.7 (4.2)	0.835	14.1 (3.8)	0.938	15.2 (3.9)	0.371	15.6 (3.2)	0.625	14.7 (3.7)	0.705	11.7 (4.8)	0.885
	Noncritical	12.6 (4.5)		14.1 (4.1)		15.5 (3.5)		15.8 (3.4)		15.8 (3.4)		14.5 (4.3)	

Experience	≤ 20	12.4 (4.3)	0.002	13.9 (4.1)	0.018	15.3 (3.7)	0.028	15.8 (3.3)	0.637	14.3 (4.1)	0.001	11.7 (5.0)	0.225
	> 20	14.4 (5.0)		15.3 (3.7)		16.5 (3.3)		16.0 (3.7)		16.3 (3.9)		12.5 (5.5)	

Smoking	No	12.8 (4.4)	0.140	14.1 (4.0)	0.648	15.5 (3.6)	0.918	15.7 (3.4)	0.426	14.7 (4.1)	0.246	11.9 (4.9)	0.387
	Yes	12.0 (4.5)		13.9 (4.1)		15.4 (3.8)		16.0 (3.3)		14.1 (4.3)		11.4 (5.3)	

Marital status	Single	12.7 (4.3)	0.871	14.3 (4.1)	0.659	15.5 (3.7)	0.681	16.1 (3.1)	0.416	14.7 (3.8)	0.678	12.3 (4.3)	0.260
	Married	12.7 (4.4)		14.1 (3.9)		15.5 (3.7)		15.6 (3.6)		15.2 (4.3)		11.9 (5.2)	

Educational level	Diploma	12.6 (4.8)	0.844	13.9 (4.4)	0.658	15.5 (3.8)	0.649	15.9 (3.5)	0.727	14.7 (4.5)	0.743	11.7 (5.5)	0.515
	BSc	12.6 (4.2)		14.2 (3.8)		15.3 (3.6)		15.7 (3.4)		14.4 (3.9)		11.7 (4.6)	
	MSN or PhD	13.0 (3.9)		14.5 (3.7)		15.9 (3.1)		16.1 (3.0)		14.5 (3.9)		12.7 (4.5)	

BMI	Underweight	12.4 (4.6)	0.657	15.1 (6.3)	0.606	14.6 (3.2)	0.793	14.8 (3.0)	0.527	15.1 (3.9)	0.423	12.3 (6.5)	0.676
	Normal	12.6 (4.3)		14.1 (4.0)		15.6 (3.7)		16.1 (3.2)		14.7 (3.9)		12.1 (4.8)	
	Overweight	12.4 (4.5)		13.8 (4.0)		15.3 (3.7)		15.6 (3.4)		14.2 (4.2)		11.5 (5.1)	
	Obesity	13.1 (4.5)		14.4 (4.0)		15.5 (3.5)		15.7 (3.6)		15.0 (4.5)		11.6 (5.2)	

**Table 6:** The correlation between distraction (minutes per week) and flourishing domains.

**Distraction/domains**	**Domain 1**	**Domain 2**	**Domain 3**	**Domain 4**	**Domain 5**	**Domain 6**
*r*	−0.172	−0.202	−0.076	−0.036	−0.142	−0.048
Sig	−0.001	−0.001	−0.112	−0.456	−0.003	−0.313

*Note:* Domain 1: Happiness and Life satisfaction, Domain 2: Mental and Physical Health, Domain 3: Meaning and Purpose, Domain 4: Character and Virtue, Domain 5: Close Social Relationship, and Domain 6: Financial and Material Stability.

**Table 7:** Regression analysis results for predicting distraction.

**Predictor**	**B**	**SE**	**β**	**Sig**	**R^2^**	**F**	**Adj R^2^**
Intercept	927.734	111.356	—	0.001	0.104	12.537	0.096
Experience	−16.299	3.766	−0.198	0.001			
Mental and Physical Health domain	−29.009	7.090	−0.187	0.001			
Smoking (ref: no)							
Yes	165.111	70.547	0.107	0.020			
Educational level (ref: diploma)							
MSN or PhD	222.144	98.941	0.103	0.025			

## Data Availability

The data that support the findings of this study are available on request from the corresponding author.
